# Respiration rate and volume measurements using wearable strain sensors

**DOI:** 10.1038/s41746-019-0083-3

**Published:** 2019-02-13

**Authors:** Michael Chu, Thao Nguyen, Vaibhav Pandey, Yongxiao Zhou, Hoang N. Pham, Ronen Bar-Yoseph, Shlomit Radom-Aizik, Ramesh Jain, Dan M. Cooper, Michelle Khine

**Affiliations:** 1 Department of Biomedical Engineering, University of California, Irvine, CA USA; 2 Department of Chemical Engineering, University of California, Irvine, CA USA; 3 Bren School of Information and Computer Sciences, University of California, Irvine, CA USA; 4 Pediatric Exercise and Genomics Research Center, School of Medicine, University of California, Irvine, CA USA; 5 Department of Pediatrics, Rambam Medical Center, Haifa, Israel

**Keywords:** Health care, Biomedical engineering

## Abstract

Current methods for continuous respiration monitoring such as respiratory inductive or optoelectronic plethysmography are limited to clinical or research settings; most wearable systems reported only measures respiration rate. Here we introduce a wearable sensor capable of simultaneously measuring both respiration rate and volume with high fidelity. Our disposable respiration sensor with a Band-Aid© like formfactor can measure both respiration rate and volume by simply measuring the local strain of the ribcage and abdomen during breathing. We demonstrate that both metrics are highly correlated to measurements from a medical grade continuous spirometer on participants at rest. Additionally, we also show that the system is capable of detecting respiration under various ambulatory conditions. Because these low-powered piezo-resistive sensors can be integrated with wireless Bluetooth units, they can be useful in monitoring patients with chronic respiratory diseases in everyday settings.

## Introduction

Chronic respiratory disease (CRD) is a growing global health and economic burden. Two common CRDs, asthma and chronic obstructive pulmonary disease (COPD), affect over 435 million people worldwide;^1^ moreover, they each have an estimated medical cost of 50 billion dollars per year.^2,3^ Fortunately, most CRDs can be well controlled or even cured with proper monitoring and care.^1^ Patients with a CRD should be mindful of their respiratory status, and any sudden changes in condition should be addressed immediately to prevent further exacerbations.^4^

There are several methods for assessing general respiratory health. The most common methods are pulmonary function tests (PFTs) that range from simple spirometry, which can be used to assess a patient’s airflow,^5,6^ to full body plethysmography^7^ used to assess lung volumes. Other methods include arterial blood sampling and diffusion capacity.^8^ While these evaluations are effective in assessing a patient’s respiratory health at a specific point in time in a laboratory setting, they cannot continuously monitor a patient’s respiratory state under normal daily environments. Moreover, PFTs such as spirometry require the patient to breathe maximally into a mouthpiece, a maneuver that is challenging, which makes these types of tests difficult to ensure accurate readings and are not suitable for long term use.

Within a clinical setting, continuous monitors can be used to track a patient’s respiration so that any measured changes in breathing patterns can be used as markers for intervention or as data for diagnoses.^9,10^ Apart from intervention and diagnosis purposes, recent studies have also shown that data acquired from continuous respiration can provide valuable information on a patient’s respiratory health and recovery.^11^ Continuous respiration monitoring can be achieved through different methods. Respiratory inductive plethysmography (RIP) uses two inductive belts placed around the abdomen and ribcage to measure the changes in circumference during respiration.^12,13^ The respiration volume can be calculated by knowing the change in circumference of both locations. This concept was first developed by Kono and Mead in 1967 and has since been well established for monitoring patients in a clinical setting.^14–17^ However, because the bands are bulky and prone to slippage, this technology does not lend itself to monitoring patients throughout the day in their native environments.^18^ Similar to RIP, the motion of the chest wall and abdomen can also be measured visually using cameras or depth sensors. Optoelectronic plethysmography (OEP) uses several cameras to monitor reflective markers placed on the torso of the subject.^19,20^ The 3D coordinates of each marker can be determined, and a topographic map of the torso can be generated over time. The change in the topography can then be used to calculated respiration volume and rate. A much higher resolution topography of the torso can also be generated using depth sensors, such as the Kinect,^21,22^ to calculate respiration volume and rate. Transthoracic impedance measurements have also been used to calculate respiration rate and volume by measuring the change in impedance of the torso between several electrodes during respiration.^23^

While these methods can all accurately track respiration rate and volume, they are either cumbersome to wear or require constant line of sight access to the patient’s entire torso, which limits their use to research or clinical settings. Researchers have developed modified RIP systems that are more portable; however, the devices are still large and cumbersome as RIP inherently requires access to the entire circumference of the chest and abdomen.^24,25^ Active monitoring of a patient’s vitals requires the device to move seamlessly with the patient and to have an unobtrusive wearable form-factor. Wearable respiration monitors developed for these purposes are therefore small, and discrete, making application and wear easy for the patient.^26–28^ However, the sensors reported in literature only measure respiration rate, and not volume. Mechanical based sensors, such as strain and capacitive, have been developed to record torso movement to calculate respiration rate.^29–31^ Researchers have also developed acoustic based sensors to listen to the air moving through the airway,^32^ and the actual breath itself can also be monitored using breath sensors placed under the nose.^33^ There is currently, to our knowledge, no unobtrusive system with a wearable form factor that can measure both respiration volume as well as respiration rate.

**Fig. 1:**
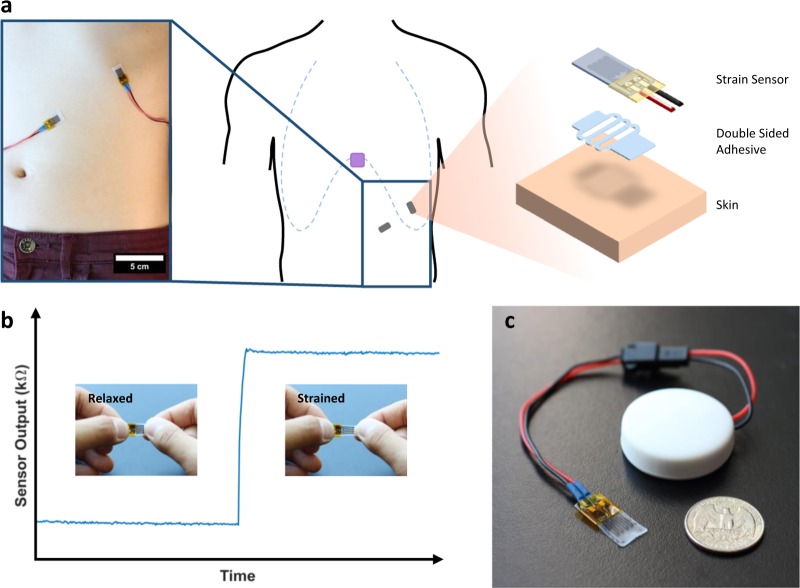
**a** The left image shows the strain sensors on the ribcage and abdomen. The middle schematics shows the placement of the accelerometer (purple square) in addition to the strain sensors (gray rectangles). The exploded schematic on the right shows the strain sensor and double-sided tape in order of attachment on the skin. All subject test was conducted using a wired data acquisition unit (Supplementary Figure 1). **b** Change in resistance of the sensor, under strain, measured using the wireless Bluetooth unit. **c** Image of the wireless Bluetooth unit with a single strain sensor attached

In this paper, we demonstrate that it is possible to measure both respiration rate and volume using a disposable wearable strain sensor placed discreetly on the abdomen and ribcage (Fig. 1a). The respiration sensor itself has a footprint smaller than that of a typical Band-aid© and only measures the local change in strain on the respective locations of the torso. Based on these measurements, we demonstrate that respiration volume and rate can still be calculated using the principles developed by Kono and Mead.^14^ A calibration model was created for each subject to calculate respiration volume and substantial agreement with the gold standard spirometry was achieved. Additionally, we also show initial proof of concept that the sensors can still record respiration signals under walking and running conditions. While all subject tests were conducted using a tethered data acquisition unit to ensure accurate data alignment (Supplemental Fig. 1), we also demonstrate that wireless respiration monitoring is achievable using a small Bluetooth module (Fig. 1b, c). The high-fidelity signals, small form-factor, and wireless capability makes continuous monitoring of respiration rate and volume in daily environments feasible.

## Results

### Strain Sensor Characteristics and Testing Setup

The type of strain sensors used in this study was previously reported by Pegan et al.; the sensor itself is composed of a piezo-resistive metal thin film set in a silicone elastomer substrate.^34^ The sensing mechanism is based on controlled fracturing of the metal thin film to increase resistance with respect to strain. The thin film itself has integrated hierarchal (nano- and micro- sized) wrinkle structures that not only act as strain relieving features but also help control the crack propagation, allowing the sensor to have a greater dynamic range while maintaining sensitivity.^34^ The sensor design used in this study has a maximum range of 156%–226% strain and has been cycled up to 2000 cycles (Supplemental Fig. 2). While there is a large spread in the maximum strain between the sensors, the failure point is still far above the range for measuring respiration. The sensors specifically used for the subject tests have linear responses (*R*^2^ between 0.96 and 1.0) with gauge factors ranging from .85 to 2.64 when taken up to 40% strain (Supplemental Fig. 2).

Similar to RIP, the strain sensors were applied on the ribcage and abdomen to measure the expansion and contraction of the respective locations during respiration. The sensors have small footprints, with a dimension of 21 mm by 10 mm by .5 mm and were placed perpendicular to each other to minimize crosstalk. Double-sided, FDA approved, adhesive was used to adhere the sensors to the skin; however, because the tape itself is not inherently stretchable, strain relief patterns were cut into the tape to allow the sensor to stretch with the skin. The ends of the sensors were adhered onto the skin, and a single strip of double-sided adhesive was placed widthwise in the center of the sensor to prevent it from completely lifting off the skin when compressed. This allowed the sensor to fold and stretch with the skin (Supplemental Fig. 3). The ends of the sensor and wires were secured with medical tape.

**Fig. 2:**
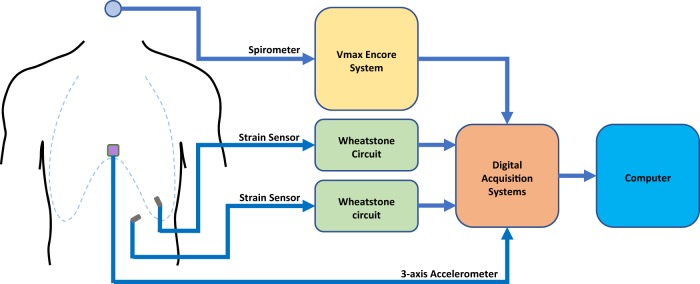
Schematic of the hardware setup for the human subject test. The spirometer (blue circle) was placed in the mouth and held in place using a strap; a nose plug was used to prevent breathing through the nose. The strain sensors (gray rectangles) were placed perpendicular to each other on the chest and abdomen. The accelerometer (purple square) was placed right below the sternum. Airflow was measured by the spirometer and processed by the Vmax Encore system; the data was then outputted in real time to one of the analog inputs on the digital acquisition system. Two Wheatstone bridges were used to calculate resistance using 4.7 kΩ resistors. The output from the accelerometer was directly measured by the digital acquisition system

All subjects recruited for this study were healthy individuals with no active respiratory problems. Subject 1 had moderately severe asthma during childhood, but no symptoms were present during the study. The group had an average height of 172 cm with a standard deviation of 8 cm, an average weight of 65 kg with a standard deviation of 10 kg, and an average BMI of 22 kg/m^2^ with a standard deviation of 2 kg/m^2^. Supplemental Table 1 contains the metrics for each individual. In addition to the strain sensors, a 3-axis accelerometer was also adhered below the sternum to detect and measure any motion during all testing. The actual respiration volume was measured using a continuous spirometer secured using a head strap. Figure 2 shows a process flow of the wired data acquisition system; all the measurements made for subjects 1–8 were completed using this setup. Subjects 1–7 performed the test procedure in a reclined position to minimize motion artifact and patient discomfort; subject 8 was tested in the standing position under walking and running conditions. All subjects completed the test procedure without complications; however, subject 6 was retested due to poor sensor placement during the initial test.

The respiration sensor was also worn on the ribcage for two hours while the data was recorded wirelessly through a Bluetooth acquisition unit. The subject was asked to periodically sit and breath normally for 2 min; each interval was graphed in Supplemental Fig. 4.

### Calibration Model for Volume Measurement

**Fig. 3:**
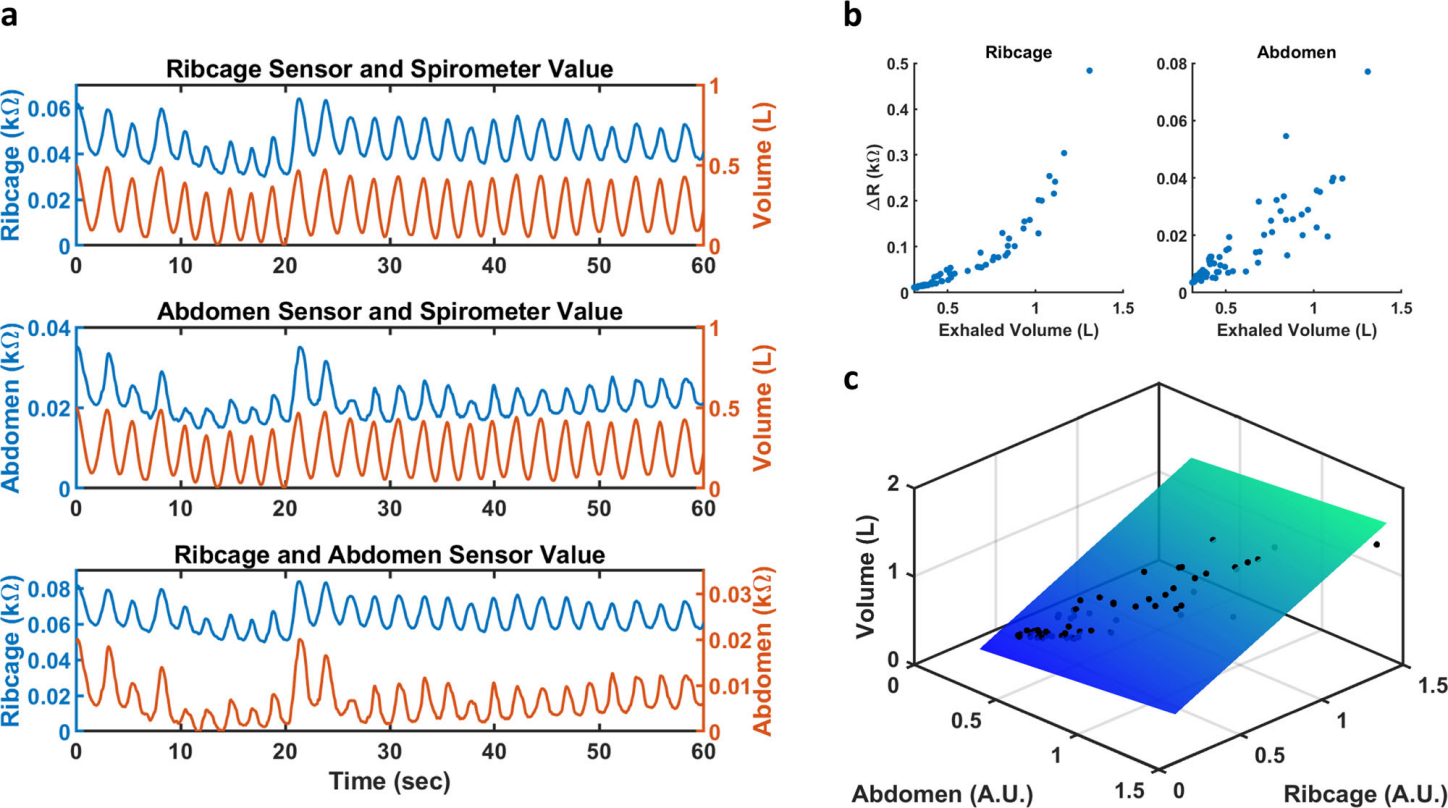
**a** Representative plots of the resistance measured by the strain sensors on the ribcage (top) and abdomen (middle) plotted with the simultaneous respiration volume. The resistance of the sensor on the ribcage and abdomen are plotted together on the bottom graph. The waveforms are detrended and shifted for ease of viewing. **b** Scatterplot of the change in resistance (ΔR) for the strain sensors on the ribcage (left) and abdomen (right) plotted against exhaled volume. **c** 3D scatterplot of the transformed ΔR for the abdomen and ribcage plotted against exhalation volume; the best fit plane is also shown

In order to calculate respiration volume from the strain sensors, a calibration model was first developed between the respiration volume and strain sensor’s output for each individual. The continuous respiration volume was measured for each subject while the expansion and contraction of the ribcage and abdomen were concurrently recorded using the strain sensors. The breath by breath exhalation volume and associated change in resistance (ΔR) of the strain sensors were then calculated and used to build the calibration model. To ensure a comprehensive model, a wide range of respiration volumes were measured; each subject was instructed to breath at three different depths (shallow, medium, and deep) at their discretion since lung capacity varies between individuals. Figure 3a shows a representative waveform of the sensor’s output plotted with respiration volume; a change in breathing amplitude occurred at the 20 s mark. Figure 3b shows a representative scatterplot of the breath by breath exhalation volume and the ΔR of the strain sensors on the abdomen and ribcage.

The relationship between the ΔR of the strain sensors and the exhalation volume generally followed a power regression model for all subjects (Supplemental Table 2). Separate power models were fitted for the abdomen and ribcage sensor values of each person; the ΔR of each sensor was then linearized using their respective power model. Afterwards, Multiple linear regression (MLR) was applied to find the best fit plane between the transformed ΔR and the exhaled volume;^35^ Fig. 3c shows a representative scatterplot of the transformed ΔR and the exhalation volume with the best fit plane plotted. Table 1 lists the adjusted *R*^2^ values as well as the standard error estimation (SEE) for the power fit and multiple linear regression. In general, the *R*^2^ value from the power fit of the abdomen showed more variations compared with the ribcage; this is also reflected in the SEE, with the abdomen having comparatively much higher values. Compared to their respective power models, the adjusted *R*^2^ and SEE reported for the MLR showed an overall improved fit for each individual with higher *R*^2^ values (all 0.92 or higher) and lower SEE (all 0.213 or lower). It should also be noted that for all subjects, one of the two power models can already account for the majority of the exhalation volume, with respect to the ΔR of the strain sensor, nearly as well as the MLR model. However, the location of the better fit is not always consistent between subjects, making it difficult to rely on a single location to calculate respiration volume. Thus, to create a comprehensive model that is not location dependent, both the ribcage and abdomen was accounted for in the MLR; however, it could be conceivable to only use one sensor, if the patient’s respiration is consistently and dominantly captured through one modality.

**Table 1:** *R*^2^ values and SEE for the power and multiple linear regression model of each subject

		Power Regression (Ribcage)		Power Regression (Abdomen)		MLR (Ribcage + Abdomen)	
Subject	Sex	*R* ^2^	SEE	*R* ^2^	SEE	*R* ^2^	SEE
1	Male	0.92	0.122	0.87	0.151	0.93	0.111
2	Male	0.83	0.2	0.9	0.15	0.96	0.098
3	Female	0.91	0.226	0.67	0.426	0.92	0.213
4	Female	0.97	0.173	0.76	0.462	0.97	0.166
5	Male	0.83	0.236	0.94	0.136	0.94	0.136
6	Female	0.96	0.054	0.8	0.12	0.96	0.051
7	Male	0.95	0.166	0.94	0.186	0.97	0.139

### Agreement between respiration volume and respiration rate

**Fig. 4:**
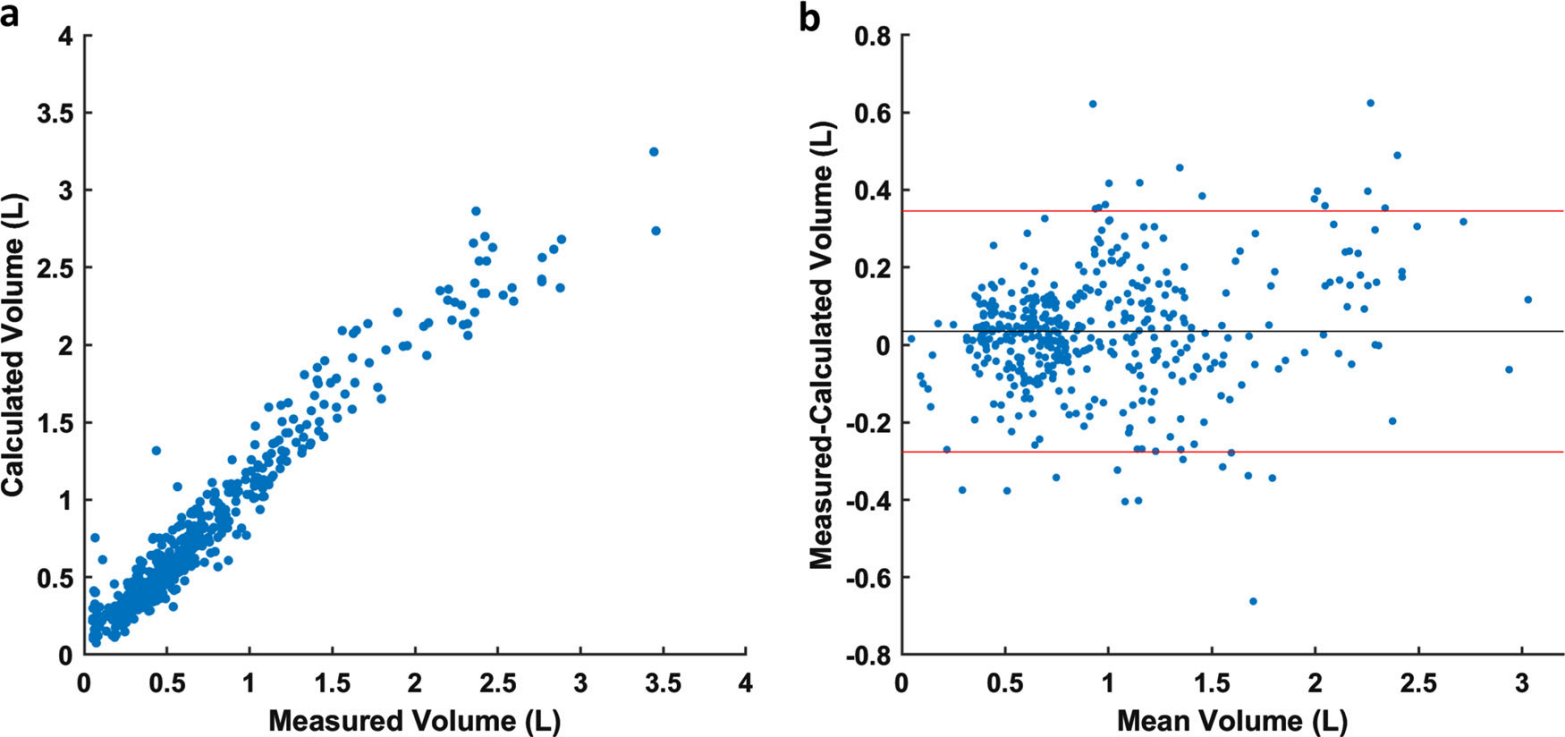
**a** Scatterplot of the calculated exhalation volume plotted against the measured exhalation volume for all subjects from the test set. **b** The Bland Altman plot of the measured exhalation volume and the calculated exhalation volume. The black line indicates the bias, and the red lines indicates the limit of agreements

To determine the fidelity of the strain sensor measurement and calibration model, each subject was instructed to breath at different volumes in random order so that a test dataset can be created. The breath by breath exhalation volume and associated ΔR of each sensor was first linearized using the respective power model from the calibration step; afterwards, MLR model was used to calculate the exhalation volume. Figure 4a shows the scatterplot of the calculated and measured exhalation volume for all subjects; Fig. 4b is the corresponding Bland Altman.^36^ The combined data, across all subjects had a bias of −.077 l with limit of agreements (LoAs) of −0.374 l and .220 l. Supplemental Table 3 contains the biases and LoAs for each individual. More importantly, there was substantial agreement between the measured and calculated exhalation volume with a concordance correlation coefficient^37^ of 0.962; the measured and calculated inhalation volume had a moderate agreement with a concordance correlation coefficient of .929.

**Fig. 5:**
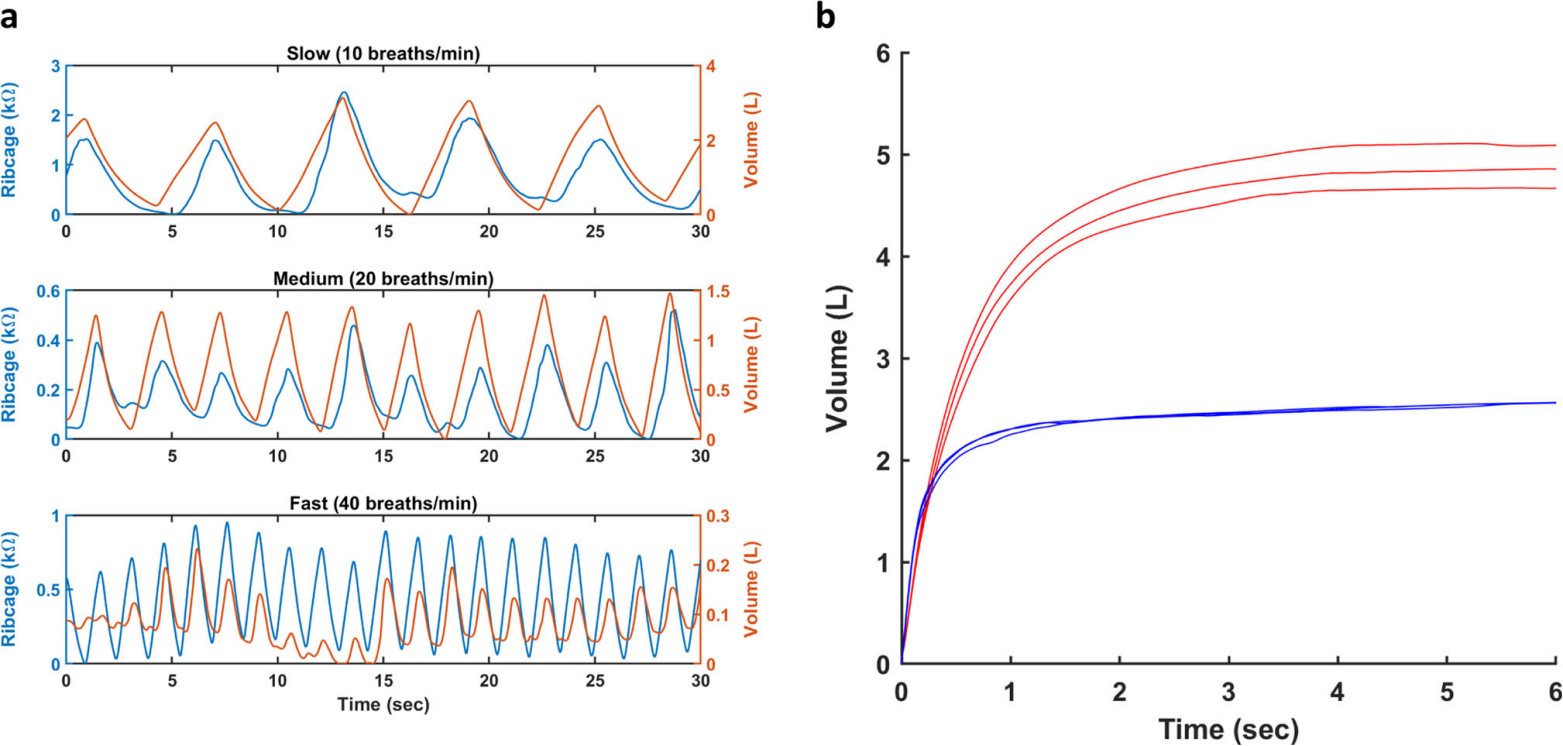
**a** Representative detrended waveforms from the ribcage strain sensor and the measured respiration volume for breathing paced at different frequencies. **b** Representative plots of the calculated (blue) and measured (red) forced exhalation volume for the spirometry PFT; the top 3 maneuvers, as determined from the measured volume based on standards set by the ATS,^6^ are plotted

To determine the sensor’s ability to measure respiration rate, the subject was asked to pace their breathing with a metronome at 10, 20, and 40 breaths per minute. Figure 5a shows a representative waveform of the detrended respiration volume and resistance measured from the ribcage strain sensor for different rates. The peak to peak period for each respiration rate was calculated and averaged for the spirometer and each strain sensor. A one-way analysis of variance (ANOVA) found no significant differences between periods calculated from the strain sensors and spirometer across all respiration rate for each subject. The averaged period and p-value for each subject is listed in Supplementary Table 4. Additionally, the respiration volume was also calculated for the paced dataset. The bias between the measured and calculated volume for all subject combined was .0347 l with LoAs of −.276 l and .346 l. There was also substantial agreement between the measured and calculated exhalation volume with a concordance correlation coefficient of .956; the inhalation data had moderate correlation with a concordance correlation coefficient of .924.

The strain sensor’s ability to measure standard spirometry was also examined for subjects 1–7. A standard spirometry maneuver requires the patient to inhale maximally and exhale forcefully with a sustained exhalation of 6 s. Each subject was asked to perform this maneuver 5 times with breaks in between; the calibration model was then applied to the ΔR of the strain sensors over time, with respect to the exhalation start, to calculate the forced expiratory volume at one second (FEV1) and forced vital capacity (FVC), and ratio of FEV1 to FVC (FEV1%); Supplementary Table 5 shows the FVC, FEV1, and FEV1% for each individual. Figure 5b shows a representative waveform the top 3 spirometry PFT for the measured and calculated volume. All subjects had calculated FEV1 and FVC values that were lower than the measured volume, indicating that the strain sensors were not able to accurately measure volume during large and forced exhalations. However, the calculated FEV1% between the measured and calculated volumes are relatively closer, but not all statistically insignificant, suggesting that a rough ratio may be maintained.

### Respiration During Motion

In order to understand the sensor’s performance under motion, subject 8 was asked to perform similar respiration procedures while walking at 4.8 km/h; additionally, he was also asked to run at 9.7 and 12.9 km/h as well. The strain sensors and accelerometer placement were kept consistent with subjects 1–7, and respiration volume was also measured concurrently.

**Fig. 6:**
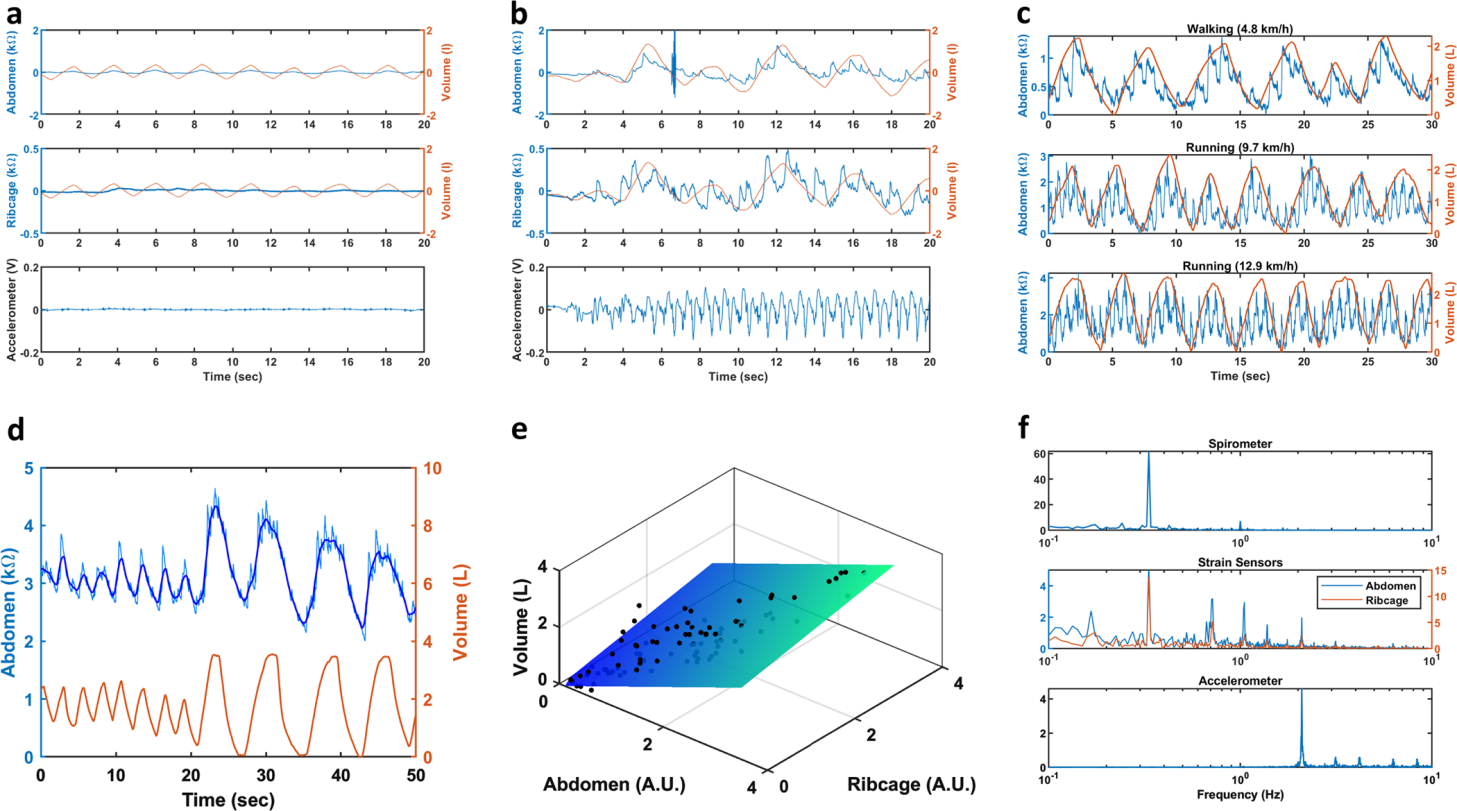
**a** Plots of the detrended output from the abdomen and ribcage strain sensors, spirometer, and accelerometer while the subjection is standing stationary. **b** Plots of the detrended outputs as the subject started walking; the respiration volume slowly increases as the subject reaches a steady state walking speed. The artifact just past 6 s is interference caused by an analog event marker used to identify the end of the speed ramping for the treadmill. **c** Plots of the detrended unfiltered signal from the abdomen strain sensor for walking at 4.8 km/h, running at 9.7 km/h, and running at 12.9 km/h. The concurrent detrended respiration volumes are plotted as well. **d** Representative plots of the raw (light blue) and filtered (blue) signal from the abdomen strain sensor and measured respiration volume during walking. The waveforms are detrended and shifted for ease of viewing. **e** 3D scatterplot of the transformed ΔR for the abdomen and ribcage strain sensors plotted against the exhalation volume during walking. **f** Plots of the frequency domain for the respiration volume measured from the spirometer (top), resistance measured from the abdomen and ribcage strain sensors (middle), and voltage measured from the y-component of the accelerometer (bottom), during the paced respiration while walking. *Y*-axis is in arbitrary units

Subject 8 performed the same calibration and test procedure as subjects 1–7 while walking. Despite being under motion, the strain sensors were still able to measure the displacement of the ribcage and abdomen during respiration. An increase in the subject’s respiration volume, from standing to walking, was also captured (Fig. 6a, b); this should be expected since the body naturally increases air intake during physical activities. Figure 6c shows the sensor signal compared to the respiration volume for the different walking and running paces.

A low pass filter was used to remove the motion artifact, which has a higher frequency, from the strain sensor data. Figure 6d shows a representative waveform of the abdomen’s strain sensor resistance before (light blue) and after (dark blue) filtering. The filtered signals were then used to calibrate the power and MLR models for walking only. The *R*^2^ value for the power fit was .80 and .62 respectively for abdomen and ribcage strain sensors; the adjusted *R*^2^ for the multiple linear regression of the transformed ΔR from strain sensor was 0.83. Figure 6e shows the scatterplot and best fit plane for the multiple linear regression. However, the calculated volume from the test set had poor agreement, with a concordance correlation coefficient of 0.75.

In addition to calculating volume, the ability to measure respiration rate while walking was also examined; the subject paced his respiration to a frequency of 20 breaths per minute while walking at 4.8 km/h. Figure 6f shows the fast Fourier transform (FFT) of the respiration volume, strain sensor resistance, and the y component of the accelerometer output during the procedure. The frequency decomposition of the strain sensor data showed matching frequency peaks with the respiration volume at .333 Hz and with the accelerometer at 2.09 Hz. Calculation of the average peak to peak period for the filtered strain sensor outputs and respiration volume showed no significant differences as determined by a one-way ANOVA (*p* = .9935).

As the subject transitioned from walking to running, the motion artifacts due to the movement also increased as well; however, the strain sensors were still able to measure the movement of the torso from respiration. As seen in Fig. 6c, the overlaid signal between the volume and strain sensor on the abdomen for all three speeds have apparent shared waveforms. The FFT of the strain sensor data and the volume shared similar frequency components for respiration; the FFT of the strain sensor data and y-component of the accelerometer also shared similar frequency peaks (Supplementary Figure 5). However, the accelerometer alone does not appear to account for all the motion artifacts present in the strain sensor signal; the strain sensor data for both running paces contained frequencies not present in the accelerometer or respiration data. For example, there were prominent peaks at 1.52 and 1.55 Hz for the 9.7 and 12.9 km/h pace respectively; this is roughly half the running frequency measured by the accelerometer, which occurred at 3.05 and 3.10 Hz for the 9.7 and 12.9 km/h pace respectively. This additional frequency may be from the repetitive strain of the skin due to the torsion of the body that occurs with running; the peaks around 1.50 Hz were not as visible during walking.

## Discussion

Under sedentary conditions, we showed that the strain sensors can calculate the respiration rate and volume based on the local changes in strain around the abdomen and ribcage with high fidelity. This follows the principles set by Kono and Mead, who reported that the mechanism for respiration has two degrees of freedom: the movement of the ribcage and movement of the abdomen. The movement in both areas, for healthy individuals, should be proportional to the volume of air inhaled and exhaled, and by taking the linear combination of the change in dimension of the respective locations, respiration volume can be calculated. While most systems described in literature uses measurements made over large area of the torso, e.g. RIP or OEP, our data suggests that measurements taken over a smaller area of the abdomen and ribcage can still be correlated to respiration volume. Moreover, the movement of the left side and right side of the ribcage and abdomen should be symmetric, so the strain sensor should only need to be applied on one side of the torso. In general, any wearable strain sensor with a sufficient gauge factor can also be applied in the same manner to calculate respiration volume.

While the breathing data taken while moving is still preliminary, it demonstrates that the respiration signal in the strain sensor is still present under motion. A calibration model was still established while walking, and respiration rate was also measured accurately. However, while a calibration model was created, the calculated respiration volume had poor agreement with the measured volume; the strain sensors were also sensitive to additional motions and artifacts that were not accounted for, such as torsion of the torso, by the accelerometer. With the proper filtering and optimization, however, it may be possible to extract respiration volume and rate under moving conditions.

Sensor geometry and flexibility are generally important factors in wearable devices. Smaller sensors with higher flexibility have the advantage of being more discrete and comfortable, making it easier to continuously monitor a patient’s health outside of a clinical setting. However, with smaller sensors, proper placement becomes more important since different locations will change the signal acquired. For example, with subject 6, placement of the strain sensor in a more lateral position on the ribcage attenuated the signal measured and required a retest. Placement of the sensors can be based on physiological markers, such as bone structures, but care must be taken to ensure that the placement is correct and consistent. In addition to small geometry, the flexible and stretchable nature of the sensor allows it to be attached intimately with the skin; because of the close contact, the sensor was less prone to slipping, an issue that occurs with RIP systems.^18,38^ However, while good adhesion prevented unwanted sensor movements, it also made the sensors more susceptible to the local deformations and mechanics of the skin, as demonstrated in subject 8. Furthermore, as the ribcage and abdomen initially to expand during inhalation, the skin must first deform before the sensors can measure a change in distance. Consequently, the ΔR will initially be much smaller since the ribcage and abdomen must expand past a point that will start stretching the skin and sensor; after this point, the ΔR should be larger for a given change in volume since the skin is already taut. The resulting relationship should be non-linear where the change in ΔR is initially small for smaller respiration volume.

The spirometry PFT highlighted the inherent hysteresis present in our system. The hysteresis is more prominent with high rate, high amplitude motions characterized by the signal undergoing a large change in resistance, as the sensor is suddenly stretched, with a slow return to baseline, as the sensor is relaxed. For normal respiration, where the amplitude change was smaller and slower compared to the PFT, the hysteresis was minimal and could be accounted for post-processing. However, with a maneuver that involved a maximal inhalation followed by a fast exhalation over a short time span, the measured resistance from the sensor was not able to return to its original baseline in time. Consequently, the measured ΔR was smaller and the calculated volume was lower. Most elastomeric systems will have some inherent hysteresis due to the viscoelastic nature of the polymer.^39^ This hysteresis needs to be accounted for or minimized, especially when measuring rate dependent information such as forced exhaled volume over time.

There are two main limitations with this study. First, the study was done with a small and homogeneous healthy population. While they provide a good initial proof of concept, further study should focus on expanding the subject population to a larger and more heterogeneous group that represent the patient demographic of interest. The second limitation is that the majority of the testing was performed while the subjects were sedentary; they were placed in a reclined position to minimize motion artifact and to ensure comfort. However, this setup does not reflect the daily environments most patients will operate in. Therefore, we demonstrated that changes in ribcage and abdomen geometry can still be measured under ambulatory condition; however, further and more exhaustive testing needs to be done to fully understand the type of motion artifacts that will be present and the general limitation of the system under motion.

In this paper, we demonstrated that respiration volume and rate can be calculated by measuring the local strain of the ribcage and abdomen with a small wearable strain sensor. Normal respiration volume and rate was determined with good fidelity under stationary conditions. Even under ambulatory conditions, we demonstrate respiration can be measured as well. While better characterization of motion is still needed, this opens the possibility for measuring respiration outside of a clinical or controlled research environment using a smaller and more discrete wearable system. High risk patient populations, such as asthmatics and COPD patients, can thus be continuously monitored for acute changes in breathing patterns, allowing for treatment to be administered promptly.

## Methods

### Strain Sensor Fabrication

Fabrication of the disposable strain sensors closely followed the process developed previously by Pegan et al.^34^ First, a shadow mask was created by laser etching the sensor design through a one-sided adhesive film adhered onto a pre-stressed polystyrene (PS) sheet (Supplemental Fig. 6-(i). The sensor design was removed, and 5 nm of Pt and Au was then respectively deposited on the masked PS sheet using a timed magnetron sputter deposition (Supplemental Fig. 6-(ii). Next, the shadow mask was removed, and the PS sheet was placed in a convection oven at 160° Celsius for 6 min to shrink (Supplemental Fig. 6-(iii). The shrunk samples were then placed in an ethanol solution containing 5 mM of (3-mercaptopropyl) trimethoxysilane (MPTMS). The MPTMS act as a molecular glue to adhere the metal thin film to the silicone elastomer. After drying, uncured silicone elastomer (Smooth-On, Ecoflex 00-30) was spin coated onto the samples at 150 r.p.m. for 35 s (Supplemental Fig. 6-(iv). The sample was then degassed and cured overnight. Afterwards, the PS was removed by submerging the samples in a 75° Celsius acetone bath; residual PS on the metal was removed using additional acetone and toluene (Supplemental Fig. 6-(v); Supplemental Fig. 6-vi shows an inset SEM image of the surface of the wrinkle metal thin film, scale bar is 50 µm. To further increase the robustness of the sensors, the metal thin film was encapsulated in by spin coating another layer of silicone elastomer over the sensing element, leaving the pads exposed (Supplemental Fig. 6-(vii). Afterwards, ribbon cables were attached onto the pads using double-sided adhesive and carbon ink (Bare Conductive, Electric Paint), and wires were soldered onto the ribbon cables. Polyimide tape was then used to encapsulate the connection (Supplemental Fig. 6-(viii).

### Hardware Setup

All the data collected for subjects 1–8 were made using a wired data acquisition system. The resistance from the strain sensor was measured using a Wheatstone bridge configuration with 4.7 kΩ resistors. The differential potential was measured using a multifunction data acquisition system (National Instruments, USB-6003). A continuous spirometer (Vyaire, Vmax Encore 229) was used to measure respiration airflow from the patient; the data was outputted as voltage from the Vmax Encore and measured using the multifunction data acquisition system. A triple axis accelerometer (Adafruit, ADXL326) was connected to a second data acquisition system to measure movement of the subject. The digital acquisition system was connected to the computer via USB, and all data was recorded and timestamped using Signal Express (National Instrument, Signal Express 2015) at a sampling frequency of 1000 Hz. Supplemental Fig. 1 shows the sensor placement on the body and hardware setup.

### Testing procedure

8 participants (5 men, 3 women) were recruited for the study. The subjects all consented to the study and were compensated for their participation. The study was approved by the Institutional Review Board at UC Irvine; informed consent was obtained from all participants. Subject 1–7 performed all testing in a reclined position, while subject 8 performed the testing under ambulatory conditions.

The subject’s height, weight, and blood pressure were recorded before the start of the testing. They were then placed in a reclined position with their lower ribcage and abdomen exposed. The strain sensor for the ribcage was placed perpendicular to the 9th and 10th rib along the midclavicular line on the left side of the torso; the strain sensor for the abdomen was placed in the upper left quadrant of the abdomen, with the long axis of the sensor perpendicular to the long axis of the ribcage sensor. The accelerometer was placed below the sternum with the *y*-axis oriented towards the abdomen, the *z*-axis oriented into the torso, and the *x*-axis oriented towards the right side of the torso. The spirometer was secured and held in place using a head strap. Before the start of the tests, the patient was given 30 s to acclimate to the set up.

For the calibration procedure, the subjects were instructed to breathe for 1 min at a shallow amplitude, 1 min at a medium amplitude, and 1 min at a deep amplitude. The maneuvers were performed sequentially with 30 s of normal tidal breathing recorded before and after the entire sequence. The calibration procedure was performed twice; the first set was performed to acclimate the subject to the set up and to advise the subject on breathing amplitude, and the second set of data used for the calibration model.

For the paced respiration, the subject was asked to pace their breathing with an audio-visual metronome. The beats per minute of the metronome was set twice as fast as the targeted breath rate so that the subjects can inhale and exhale per beat. 30 s of tidal breathing was recorded before and after each paced breathing. Breath rates of 10, 20 and 40 breaths per minute were recorded for 2 min, 1 min, and 30 s respectively.

For the test respiration, the subject was instructed to take a series of shallow, medium, and deep breath in any order at their discretion. This was done for a total of 3 min, with 30 s of tidal breathing recorded before and after the 3-minute interval. The subject performed this procedure twice, with the second set used for analysis.

For the PFT, the subject was asked to inhale maximally and forcefully exhale, sustaining the exhalation for 6 s. This maneuver was performed 5 times, and the top 3 PFTs were calculated and selected for use in the analysis as per ATS guidelines.^6^ Subjects were given a 45 s rest period between each maneuver.

Subject 8 was tested under different walking and running conditions. All testing occurred on a treadmill while the subject was upright; sensor placement was the same as subject 1–7. The calibration procedure was performed twice while the subject was standing still and twice while the subject was walking at 4.8 km/h. The paced respiration procedure was performed with a breathing frequency of 20 breaths per minute for 2 min while the subject was walking at 4.8 km/h. The subject was also asked to perform the test set procedure twice while walking at 4.8 km/h. Lastly, the subject was asked to walk and run at 4.8, 9.7 and 12.9 km/h for 2 min each.

For the wireless data acquisition, the respiration sensor was placed on the ribcage using the same procedure for subjects 1–8. Afterwards, a voltage divider with a 4.7 kΩ resistor was used to measure the sensor resistance through a Bluetooth acquisition unit (Espurino shop, Puck.js); the data was sent to a tablet through a custom app. The subject was not restricted in terms of movement and activity but was asked to periodically (approximately every 15 min) sit and breath normally for 2 min.

### Data pre-processing

MATLAB (MathWorks, R2016b) was used to process and analyze the data. The voltage output from the Vmax system was first filtered to remove spikes in voltage; afterwards, the data was multiplied by a correction constant (2.04 l/sV) to get airflow. The constant was determined before the start of the subject test (Supplemental Figure 7). The volume was calculated by integrating the respiration airflow with respect to time. To segment the inhalation and exhalation volume, the respiration airflow was integrated between two sequential y intercept of the flow data; integration of positive flow yielded exhalation while integration of negative flow yielded inhalation volume. A cutoff volume of .01 l was used to remove any volume resulting from noise about 0.

The resistance reading from the strain sensor was first filtered using a low pass filter with a cutoff frequency of 20 Hz. Afterwards, to account for the hysteresis of the sensor, a MATLAB script was used automatically to remove large jumps in resistance due to large amplitude change. For the data acquired while walking, a low pass filter was applied to the signal from the strain sensor. The cutoff frequency of the filter was determined by the dominant frequency from the accelerometer; it was assumed that the frequency of the respiration did not match the frequency of the walking or running. Afterwards, the breath by breath peaks and value from the respiration volume was used to help determine start and end of the ΔR for the strain sensor of each breath. For the spirometry test, the data was manually segmented into the 5 tests. The FEV1, FVC, and FEV1 to FVC ratio was calculated according to the ATS standards.

### Code Availability

The MATLAB codes used in this study are available from the corresponding author upon reasonable request.

## Supplementary information

Supplemental Material

## Data Availability

The data sets generated during and/or analyzed during the current study are available from the corresponding author upon reasonable request.

## Supplementary information

**Supplementary information** accompanies the paper on the *npj Digital Medicine* website (10.1038/s41746-019-0083-3).

## References

[1] Ferkol T, Schraufnagel D (2014). The Global burden of respiratory disease. Ann. Am. Thorac. Soc..

[2] Nurmagambetov T, Kuwahara R, Garbe P (2018). The economic burden of asthma in the United States, 2008–2013. Ann. Am. Thorac. Soc..

[3] Guarascio, A. J., Ray, S. M., Finch, C. K. & Self, T. H. The clinical and economic burden of chronic obstructive pulmonary disease in the USA. *Clinecon. Outcomes Res*. **5**, 235–245 (2013). 10.2147/CEOR.S34321 PMC3694800 23818799

[4] Forum of International Respiratory Societies. *The Global Impact of Respiratory Disease* (European Respiratory Society, Sheffield, 2017).

[5] Crapo RO (1994). Pulmonary-function testing. N. Engl. J. Med..

[6] Miller MR (2005). Standardisation of spirometry. Eur. Respir. J..

[7] Criée CP (2011). Body plethysmography – Its principles and clinical use. Respir. Med..

[8] Mottram C, Ruppel G (2013). Ruppel’s Manual of Pulmonary Function Testing.

[9] Brochard L (2012). Clinical review: Respiratory monitoring in the ICU - a consensus of 16. Crit. Care.

[10] Folke M, Cernerud IL, Ekstrӧm M, Hӧk IB (2003). Critical review of non-invasive respiratory monitoring in medical care. Med. Biol. Eng. Comput..

[11] Hmeidi H (2017). Tidal breathing parameters measured using structured light plethysmography in healthy children and those with asthma before and after bronchodilator. Physiol. Rep..

[12] DalľAva-Santucci, J. & Armanganidis, A. *Pulmonary Function in Mechanically Ventilated Patients* 121–142 (Springer, Berlin, Heidelberg, 1991).

[13] Sackner MA (1989). Calibration of respiratory inductive plethysmograph during natural breathing. J. Appl. Physiol..

[14] Konno K, Mead J (1967). Measurement of the separate volume changes of rib cage and abdomen during breathing. J. Appl. Physiol..

[15] Collop NA (2007). Clinical guidelines for the use of unattended portable monitors in the diagnosis of obstructive sleep apnea in adult patients. Portable Monitoring Task Force of the American Academy of Sleep Medicine. J. Clin. Sleep. Med..

[16] Cantineau JP, Escourrou P, Sartene R, Gaultier C, Goldman M (1992). Accuracy of respiratory inductive plethysmography during wakefulness and sleep in patients with obstructive sleep apnea. Chest.

[17] Farré, R., Montserrat, J. M. & Navajas, D. Noninvasive monitoring of respiratory mechanics during sleep. *Eur. Respir. J*. 10.1183/09031936.04.00072304. 10.1183/09031936.04.00072304 15572552

[18] Caretti DM, Pullen PV, Premo LA, Kuhlmann WD (1994). Reliability of respiratory inductive plethysmography for measuring tidal volume during exercise. Am. Ind. Hyg. Assoc. J..

[19] Massaroni C (2017). Optoelectronic plethysmography in clinical practice and research: a review. Respiration.

[20] Aliverti A (2000). Optoelectronic plethysmography in intensive care patients. Am. J. Respir. Crit. Care Med..

[21] Aoki, H. *et al*. Non-contact respiration measurement using structured light 3-D sensor. *SICE Annu. Conf*. 614–618 http://ieeexplore.ieee.org/stamp/stamp.jsp?tp=&arnumber=6318511&isnumber=6318306 (2012).

[22] Soleimani V (2017). Depth-based whole body photoplethysmography in remote pulmonary function testing. IEEE Trans. Biomed. Eng..

[23] Voscopoulos C (2013). Evaluation of a novel noninvasive respiration monitor providing continuous measurement of minute ventilation in ambulatory subjects in a variety of clinical scenarios. Anesth. Analg..

[24] Wilhelm FH, Roth WT, Sackner MA (2003). The lifeshirt. Behav. Modif..

[25] Lymberis, A. & De Rossi, D. E. *Wearable Ehealth Systems For Personalised Health Management: State Of The Art And Future Challenges* (IOS Press, Amsterdam, Neatherlands 2004).

[26] Yilmaz T (2010). Detecting vital signs with wearable wireless sensors. Sensors.

[27] Khan Y, Ostfeld AE, Lochner CM, Pierre A, Arias AC (2016). Monitoring of vital signs with flexible and wearable medical devices. Adv. Mater..

[28] Patel S, Park H, Bonato P, Chan L, Rodgers M (2012). A review of wearable sensors and systems with application in rehabilitation. J. Neuroeng. Rehabil..

[29] Wang Y (2014). Wearable and highly sensitive graphene strain sensors for human motion monitoring. Adv. Funct. Mater..

[30] Laukhina E (2010). Ultrasensitive piezoresistive all-organic flexible thin films. Adv. Mater..

[31] Li Y, Samad YA, Liao K (2015). From cotton to wearable pressure sensor. J. Mater. Chem. A.

[32] Mimoz O, Benard T, Gaucher A, Frasca D, Debaene B (2012). Accuracy of respiratory rate monitoring using a non-invasive acoustic method after general anaesthesia. Br. J. Anaesth..

[33] AL-Khalidi FQ, Saatchi R, Burke D, Elphick H, Tan S (2011). Respiration rate monitoring methods: A review. Pediatr. Pulmonol..

[34] Pegan JD (2016). Skin-mountable stretch sensor for wearable health monitoring. Nanoscale.

[35] Loveridge, B., West, P., Anthonisen, N. R. & Kryger, M. H. Single-position calibration of the respiratory inductance plethysmograph. *J. Appl. Physiol. Respir. Environ. Exerc. Physiol.* **55**, 1031–1034 (1983). 10.1152/jappl.1983.55.3.1031 6629900

[36] Bland JM, Altman DG (2010). Statistical methods for assessing agreement between two methods of clinical measurement. Int. J. Nurs. Stud..

[37] Lin, L. I.-K. A concordance correlation coefficient to evaluate reproducibility. *Biometrics* **45**, 1255–1268 (1989). 2720055

[38] Whyte KF (1991). Accuracy of respiratory inductive plethysmograph in measuring tidal volume during sleep. J. Appl. Physiol..

[39] Amjadi M, Kyung KU, Park I, Sitti M (2016). Stretchable, Skin-Mountable, and Wearable Strain Sensors and Their Potential Applications: a Review. Adv. Funct. Mater..

