# Supplementary material for: Respiration rate and volume measurements using wearable strain sensors
Source: NPJ Digit Med. 2019 Feb 13;2:8. doi: 10.1038/s41746-019-0083-3 (PMC6550208; doi:10.1038/s41746-019-0083-3)
Supplement: Supplementary file 1 — Supplemental Material [file 41746_2019_83_MOESM1_ESM.pdf]

Supplementary Material

Respiration Rate and Volume Measurements using Wearable Strain Sensors

Michael Chu[a], Thao Nguyen[b], Vaibhav Pandey[c], Yongxiao Zhou[a], Hoang N. Pham[d], Ronen Bar-Yosph[d,e], Shlomit Radom-Aizik[d], Ramesh Jain[c], Dan M. Cooper[d], Michelle Khine[a,b]

[a] Department of Biomedical Engineering, UC Irvine
[b] Department of Chemical Engineering, UC Irvine
[c] Bren School of Information and Computer Sciences, UC Irvine
[d] Pediatric Exercise and Genomics Research Center, School of Medicine, UC Irvine
[e] Department of Pediatrics, Rambam Medical Center

a. b.

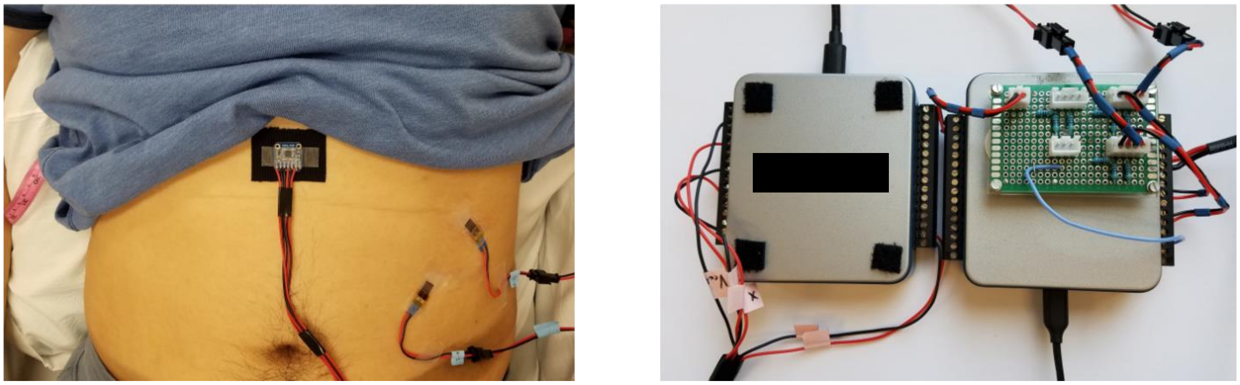

Supplementary Figure 1. (a) Image of the sensors and accelerometer placement during the stationary reclined testing. Placement during the walking and running test remained the same. (b) Image of the circuit and digital acquisition system used to record the data.

a.

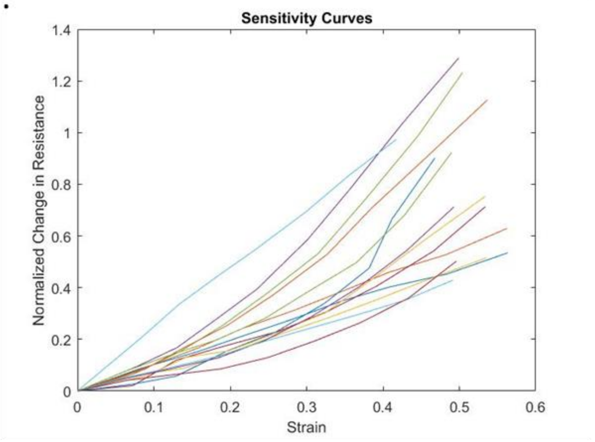

b.

Subject	Abdomen Gauge Factor	Abdomen Linear R2	Ribcage Gauge Factor	Ribcage Linear R2
1	1.79	0.97	~	~
2	1.70	0.98	1.10	0.99
3	1.02	0.94	2.34	0.97
4	1.45	0.98	1.89	1.00
5	0.97	0.99	0.97	0.98
6	1.59	1.00	2.38	0.99
7	1.07	0.99	1.46	0.96

c.

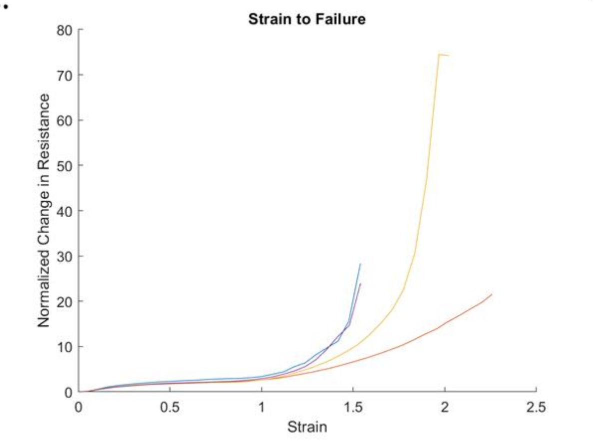

d.

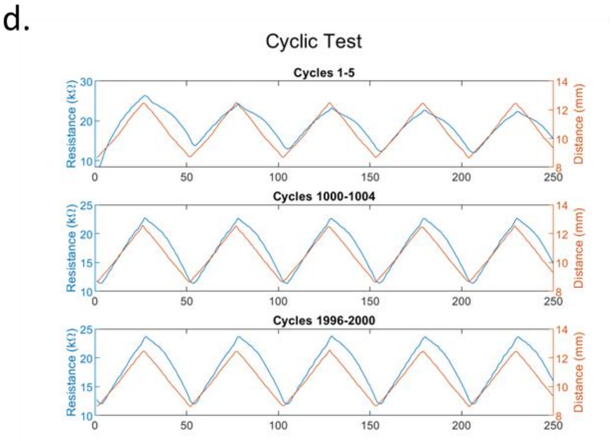

Supplementary Figure 2. (a) Graph of all the sensitivity curves for the sensors used for subject testing. (b) Table of the gauge factors and R^2 values for all sensors; a linear model was used to fit the normalized change in resistance to strain. The gauge factor and best fit was not recorded for the ribcage strain sensor for subject 1. (c) Graph of the normalized change in resistance of 4 sensors that were strained to failure; the strain the sensors failed at ranged from 156% to 226% strain. (d) Graphs of the sensor resistance during cyclic testing at 50% strain; the sensor was cycled 2000 times.

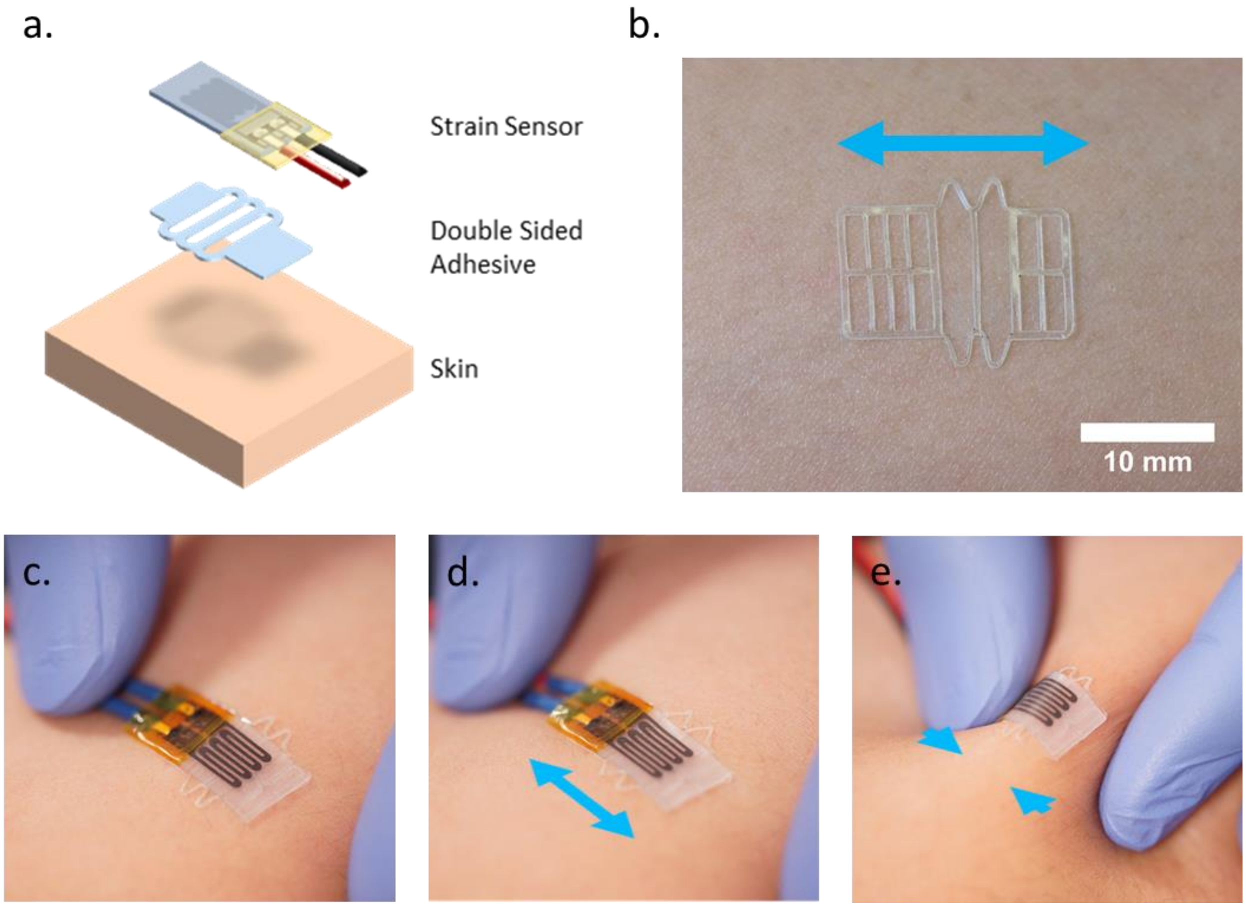

Supplementary Figure 3. (a) Exploded schematic of how the respiration sensor is adhered onto the skin. (b) Image of the double-sided tape with the curved laser cut strain relief structures. The ends of the sensor are anchored to the skin by the double-sided adhesive while a single strip of the adhesive is used to prevent the middle of the sensor from lifting off during respiration. The strain relieving structures are used to allow the sensor, and adhesive, to strain in a single axis indicated by the blue arrow. The adhesive to the skin is an FDA approved skin adhesive while the adhesive to the sensor is a silicone-based adhesive. (c-e) Images of the sensor (c) while neutral, (d) under tension, and (e) under compression. The arrow indicates direction of applied force.

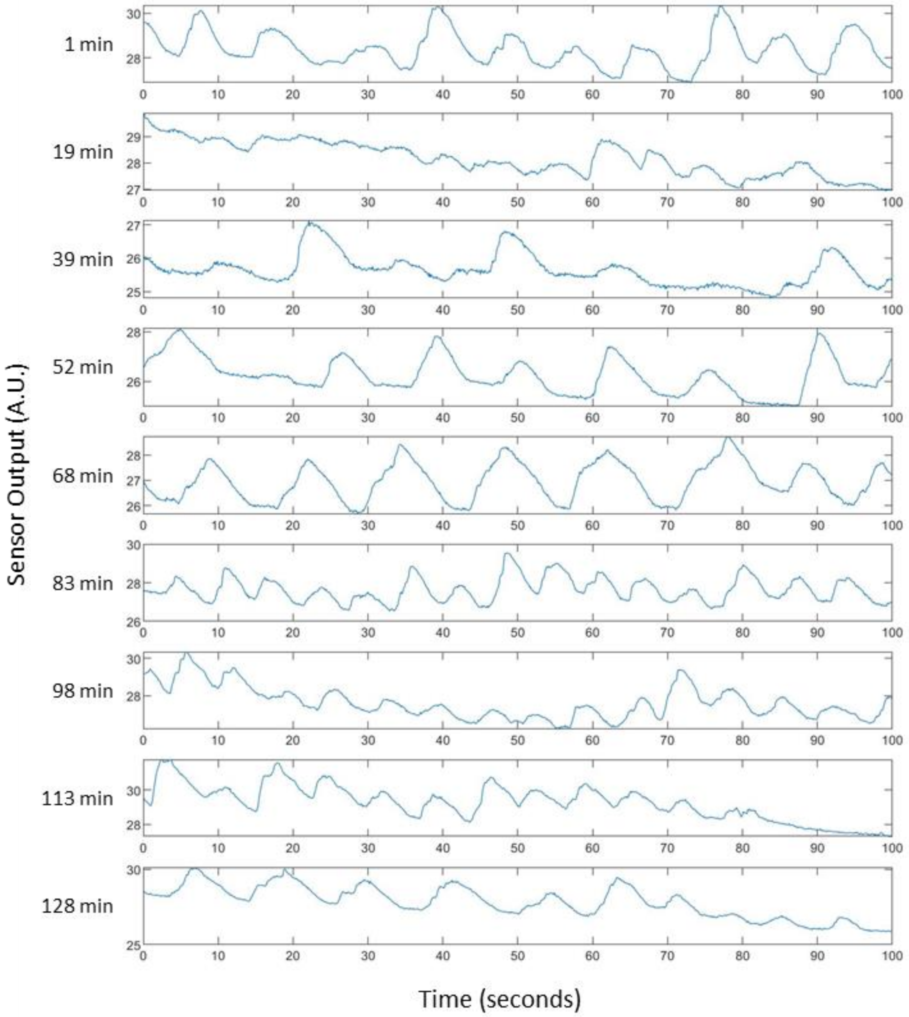

Supplementary Figure 4. Graphs of the sensor output as measured by the Bluetooth module. The subject wore the sensor continuously on the ribcage for 2 hours and was asked to periodically sit and breath normally for 2 minutes; the graph shows the sensor output while the subject was sitting.

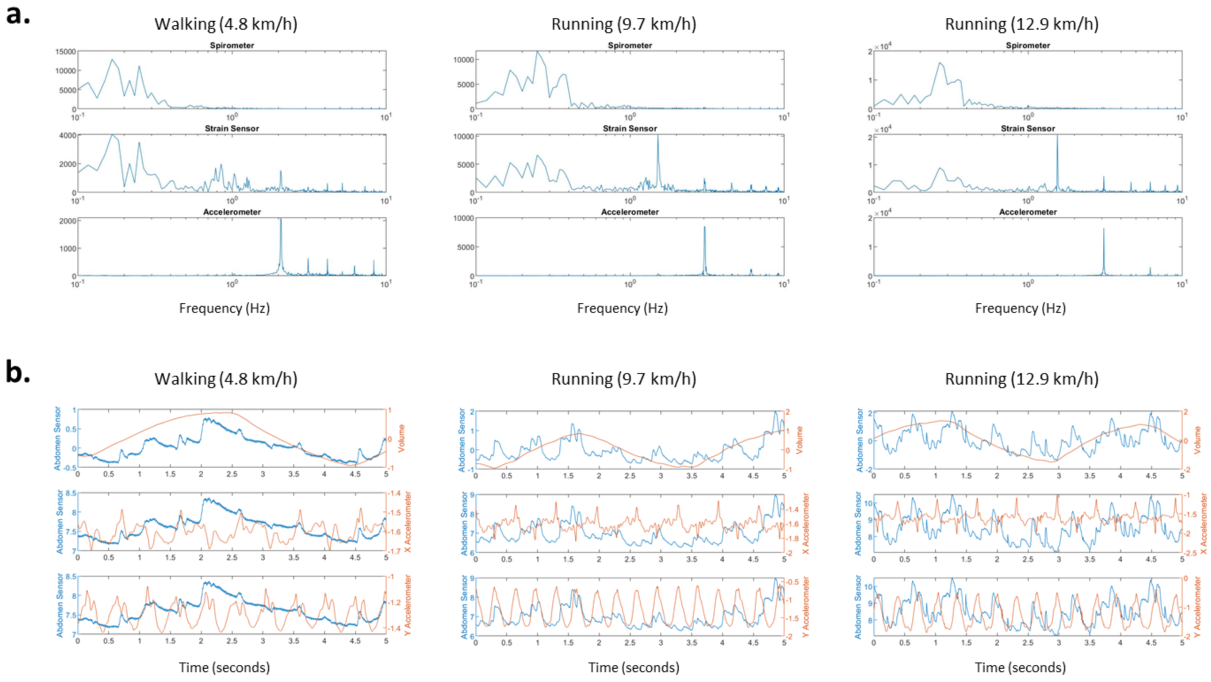

Supplementary Figure 5. (a) The frequency domain of the measured volume, abdomen strain sensor resistance, and the y-component output of the accelerometer for the walking and running exercises. (b) The waveform from the abdomen strain sensor resistance and spirometer (top), abdomen strain sensor resistance and x-axis accelerometer (middle), and abdomen strain sensor resistance and y-axis accelerometer (bottom). The x-axis is parallel to the ground and flat against the chest; the y-axis is perpendicular to the ground and flat against the body.

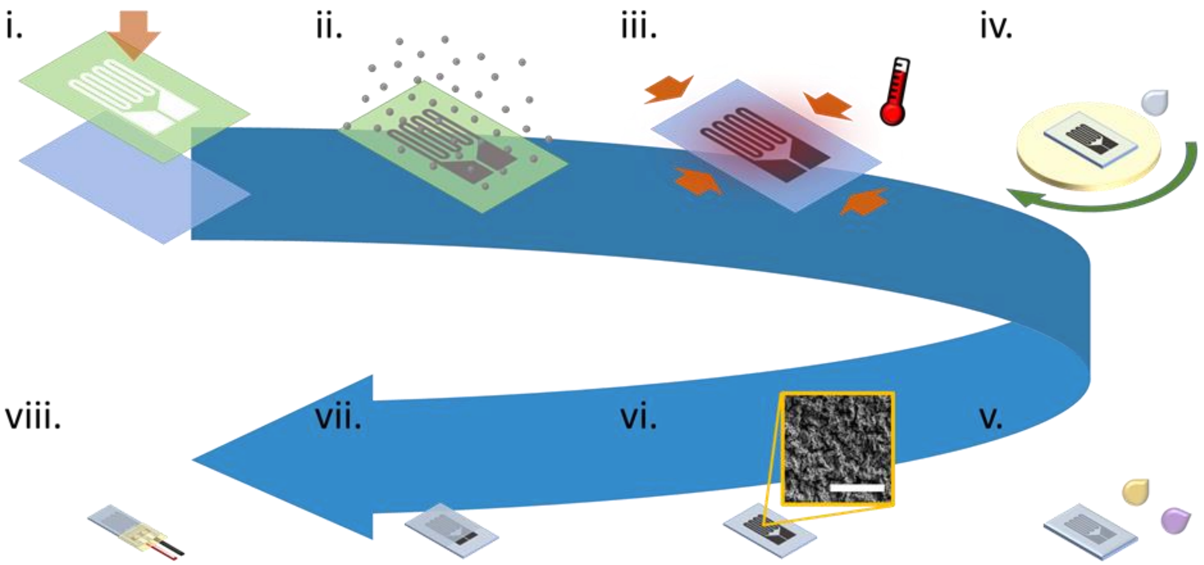

Supplementary Figure 6. Process flow of the fabrication process for the sensor. (i) Application of the shadow mask to the pre-stressed polystyrene (PS) in preparation for metal deposition. (ii) Sputter deposition of 5 nm of Pt and Au. (iii) Shrinking of the metal deposited PS in a convection oven at 160° Celsius for 6 minutes. (iv) Spin coating silicone elastomer (150 rpm for 35 seconds) after treating the sensors in an ethanol solution containing 5 mM of (3-mercaptopropyl) trimethoxysilane (MPTMS). The elastomer is cured, and the PS is removed by submerging the samples in a 75° Celsius acetone bath. (v) Residual PS on the metal was removed by washing with acetone (yellow) and toluene (purple). (vi) Scanning electron microscope (SEM) image of the wrinkled metal thin film transferred into the silicone elastomer; scale bar is 50 µm. (vii) Additional silicone elastomer spin coated on the metal thin film to increase robustness. (viii) Application of wires for connection to the data acquisition system.

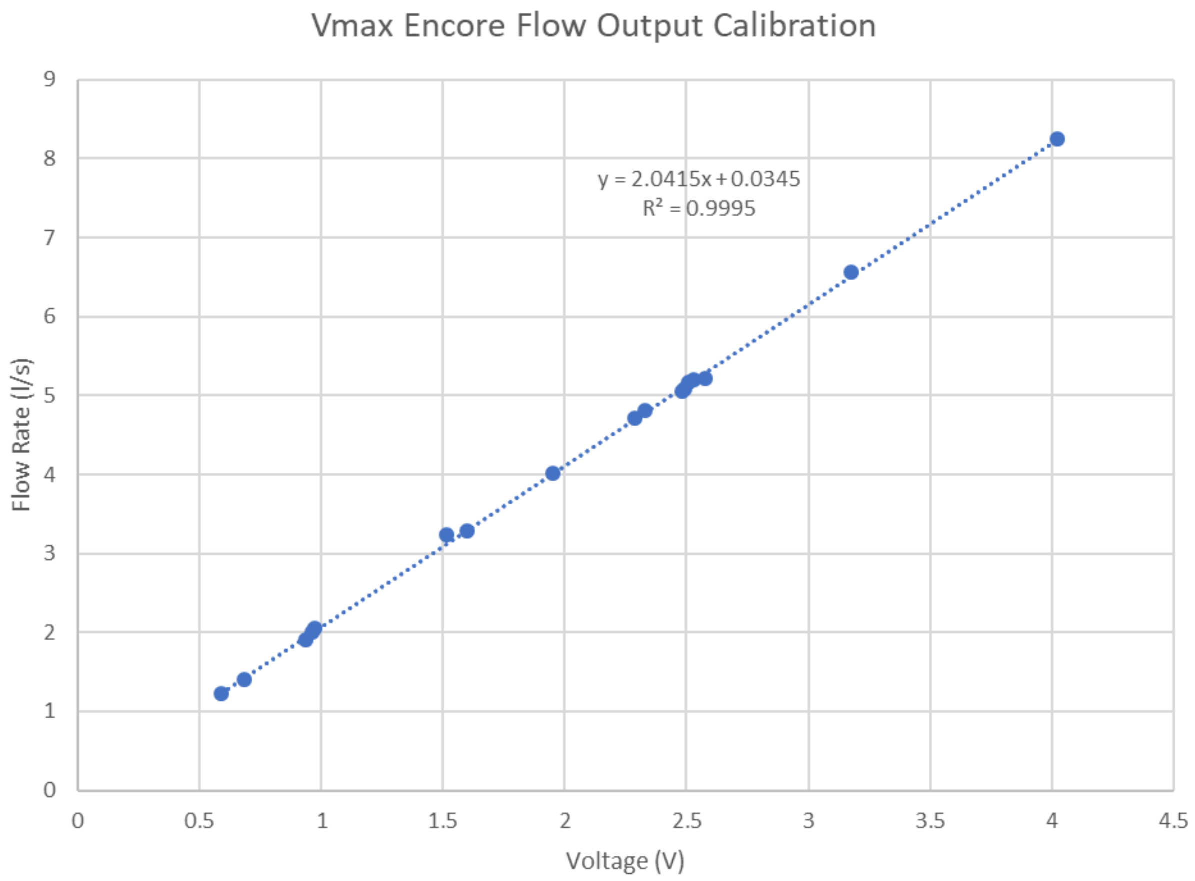

Supplementary Figure 7. Calibration curve for the respiration flow and voltage output from the Vmax Encore system.

Subject	Sex	Height (cm)	Weight (kg)	BMI (kg/m^2)
1	Male	183.5	72.8	21.6
2	Male	177.8	63.8	20.2
3	Female	162.0	54.1	20.6
4	Female	166.1	63.8	23.1
5	Male	179.4	87.1	27.1
6	Female	161.7	53.8	20.6
7	Male	177.6	68.7	21.8
8	Male	170.0	60.8	21.0

Supplementary Table 1. The sex, height, weight and body mass index (BMI) for each subject.

	Linear Fit		Power Fit	
Subject	Abdomen	Ribcage	Abdomen	Ribcage
1	0.874	0.779	0.866	0.913
2	0.913	0.676	0.902	0.826
3	0.658	0.827	0.673	0.908
4	0.536	0.866	0.759	0.966
5	0.924	0.653	0.944	0.832
6	0.739	0.816	0.800	0.959
7	0.927	0.936	0.940	0.952

Supplementary Table 2. The R^2 values for the linear and power fit of the exhalation volume and associated change in resistance (ΔR) of the abdomen and ribcage. The power regression model generally provided a better fit of the data.

Subject	Bias	Limit of Agreement	
1	-0.057	-0.316	0.202
2	0.012	-0.333	0.356
3	0.065	-0.096	0.227
4	0.066	-0.346	0.478
5	-0.054	-0.298	0.190
6	0.099	-0.158	0.356
7	0.108	-0.147	0.364

Supplementary Table 3. The bias and limit of agreements between the measured and calculated exhalation volume for the test set of each subject.

	10 breaths/min				20 breaths/min				40 breaths/min			
Subject	Spirometer	Abdomen	Ribcage	P-Value	Spirometer	Abdomen	Ribcage	P-Value	Spirometer	Abdomen	Ribcage	P-Value
1	6.02 ± .88	6.01 ± .97	6.01 ± .93	.9992	2.97 ± .21	3.00 ± .25	2.96 ± .24	.8865	1.62 ± .21	1.61 ± .28	1.62 ± .30	.9990
2	6.00 ± .21	6.00 ± .24	5.98 ± .43	.9862	2.99 ± .10	2.99 ± .11	2.99 ± .12	.9958	1.50 ± .04	1.50 ± .05	1.50 ± .04	.9877
3	6.02 ± .24	5.98 ± .53	6.02 ± .39	.9532	3.06 ± .20	3.06 ± .23	3.06 ± .20	.9999	1.50 ± .10	1.50 ± .12	1.50 ± .12	.9978
4	6.00 ± .18	6.01 ± .17	6.01 ± .18	.9935	3.00 ± .10	3.01 ± .15	3.00 ± .11	.9493	1.50 ± .07	1.47 ± .27	1.50 ± .07	.8146
5	6.01 ± .17	6.00 ± .15	6.00 ± .21	.9985	2.98 ± .13	2.97 ± .20	2.97 ± .29	.9886	1.50 ± .05	1.50 ± .08	1.50 ± .08	.9997
6	5.98 ± .32	5.98 ± .39	5.97 ± .41	.9945	3.00 ± .10	3.00 ± .28	3.01 ± .12	.9875	1.51 ± .07	1.51 ± .10	1.51 ± .09	.9779
7	6.00 ± .15	6.00 ± .15	5.99 ± .24	.9936	2.99 ± .17	2.99 ± .17	2.99 ± .17	.9988	1.50 ± .03	1.50 ± .04	1.49 ± .04	.8631

Supplementary Table 4. The averaged peak to peak period (± s.d.) from the strain sensors and spirometer data at the three different respiration rates for all subjects. The p-value from the one-way ANOVA is listed for the different respiration rate for each subject.

Subject	FEV1		FVC		FEV1%	
	Measured (L)	Calculated (L)	Measured (L)	Calculated (L)	Measured	Calculated
1	3.62 ± .04	2.19 ± .23	3.97 ± .10	2.24 ± .24	0.91 ± .01	0.98 ± .01
2	3.24 ± .11	2.08 ± .83	3.56 ± .20	2.50 ± .83	0.91 ± .02	0.83 ± .10
3	2.72 ± .03	2.25 ± .08	3.25 ± .06	2.41 ± .10	0.84 ± .01	0.93 ± .01
4	3.94 ± .05	3.67 ± .13	4.58 ± .11	4.29 ± .36	0.86 ± .03	0.86 ± .05
5	4.08 ± .01	2.40 ± .34	4.52 ± .01	2.70 ± .32	0.90 ± .00	0.89 ± .02
6	2.22 ± .00	2.03 ± .06	4.68 ± .08	2.16 ± .02	0.83 ± .02	0.94 ± .03
7	3.68 ± .17	2.27 ± .05	4.80 ± .21	2.55 ± .02	0.77 ± .00	0.89 ± .01

Supplementary Table 5. The averaged (± s.d.) calculated and measured FEV1, FVC, and FEV1% for all subjects.
